# An aggressive case of a thoracic undifferentiated SMARCA4‐deficient tumor with extensive pleural involvement

**DOI:** 10.1111/1759-7714.15230

**Published:** 2024-02-23

**Authors:** Paul F. Hanona, Daniel Ezekwudo, Joseph Fullmer, Timothy Allen, Ishmael Jaiyesimi

**Affiliations:** ^1^ Department of Hematology and Oncology Corewell Health – William Beaumont University Hospital Royal Oak Michigan USA

**Keywords:** lung cancer, SMARCA4, SMARCA4‐deficient undifferentiated thoracic tumors, undifferentiated thoracic tumors

## Abstract

SMARCA4‐deficient undifferentiated thoracic tumors are a rare phenomenon. A 40‐year‐old male was newly diagnosed with SMARCA4‐deficient undifferentiated non‐small cell lung cancer. He had a history of heavy smoking and job‐related exposure to metal dust and melted nickel. CT imaging showed numerous right‐sided pleural masses and soft tissue plaques, but no metastases. CT‐guided biopsy of a pleural mass confirmed the diagnosis. He was prescribed six cycles of carboplatin paclitaxel, and follow‐up imaging showed largely stable disease. Treatment was changed to nivolumab due to shortness of breath, and he received one cycle of nivolumab without considerable side effects. Unfortunately, during the second cycle of his nivolumab, the patient presented with new weakness. Imaging showed spinal cord metastasis and he underwent a laminectomy; he was subsequently followed up as an outpatient. The objective of this publication was to explore SMARCA4‐deficient undifferentiated thoracic tumors, other related SMARCA4‐deficient tumors, and their overall pattern of presentation. The genetic aberrations of this case are compared to recent publications that also discuss genetic aberrations commonly occurring with this disease process, with an ultimate goal of hastening detection and adding to the library of treatment results.

## Graphical abstract

SMARCA4 deficient tumors are rare and can present in multiple organ systems.This case report of a SMARCA4 deficient lung cancer exemplifies how to hasten diagnostic detection and how to navigate treatment options.

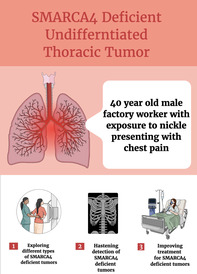

## INTRODUCTION

SMARCA4‐deficient undifferentiated thoracic tumors (SMARCA4‐UT) resulting from tumor suppressor protein inactivation, are found mostly in the anterior mediastinum, and have a median survival of 7 months.^1^, ^2^, ^3^ SMARCA4 deficient tumors have been noted to cause malignancy in the central nervous system, head and neck region, gastrointestinal tract, female genital tract, genitourinary tract, soft tissue, and thorax.^1^ Initial pathology is often determined to be sarcomatous or carcinomatous, with symptoms varying from shortness of breath to superior vena cava (SVC) syndrome.^4^, ^5^ SMARCA4‐UT should be considered in differential diagnosis once imaging shows an infiltrate that is ill‐defined, heterogeneous, large, compressive in nature, and is associated with surrounding necrosis.^6^ Currently there is no widely accepted standard of treatment for SMARCA4‐UT. Experimental treatments, discussed below, include combinations of chemotherapy, surgery, and/or radiation.^7^

## CASE REPORT

**FIGURE 1:**
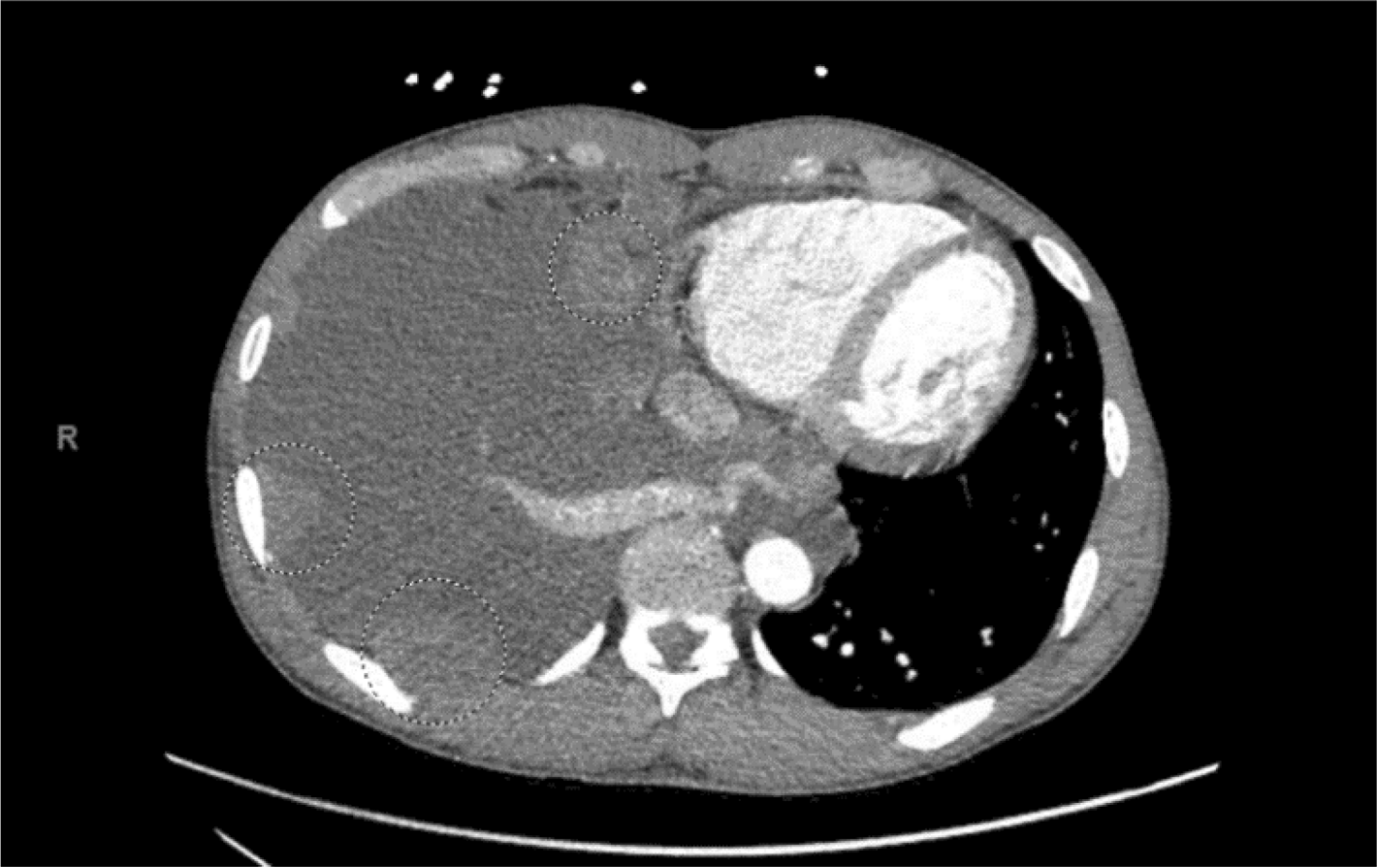
Chest computed tomography (CT) with numerous right‐sided pleural‐based masses and soft tissue plaques, and right pleural effusion.

**FIGURE 2:**
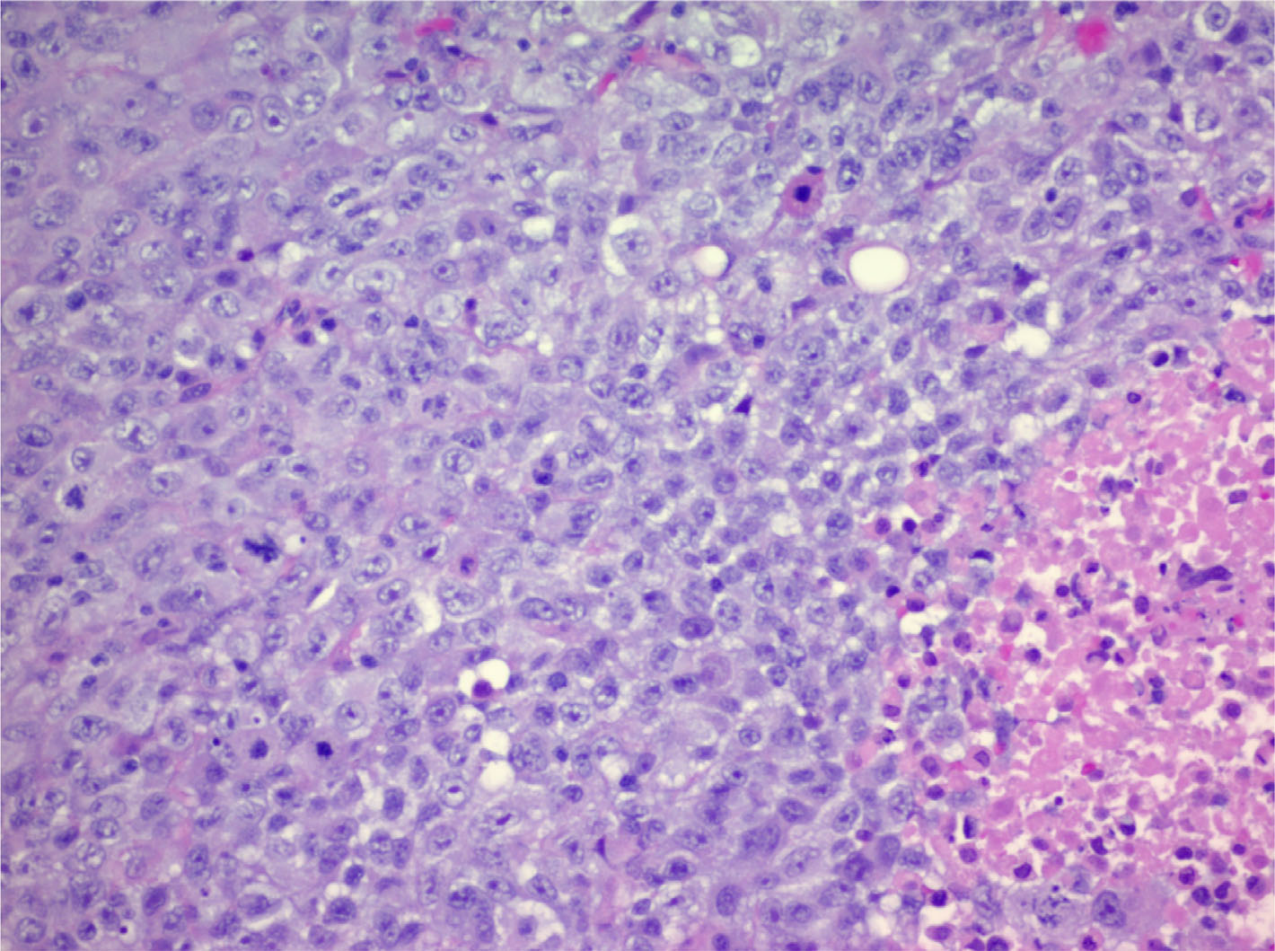
Hematoxylin and eosin (H&E) stained image of thoracic SMARCA4‐deficient undifferentiated tumor, showing sheets of round to epithelioid cells with relatively uniform nuclei and prominent nucleoli. Necrosis and mitoses, including atypical mitoses, are present. (20×).

**FIGURE 3:**
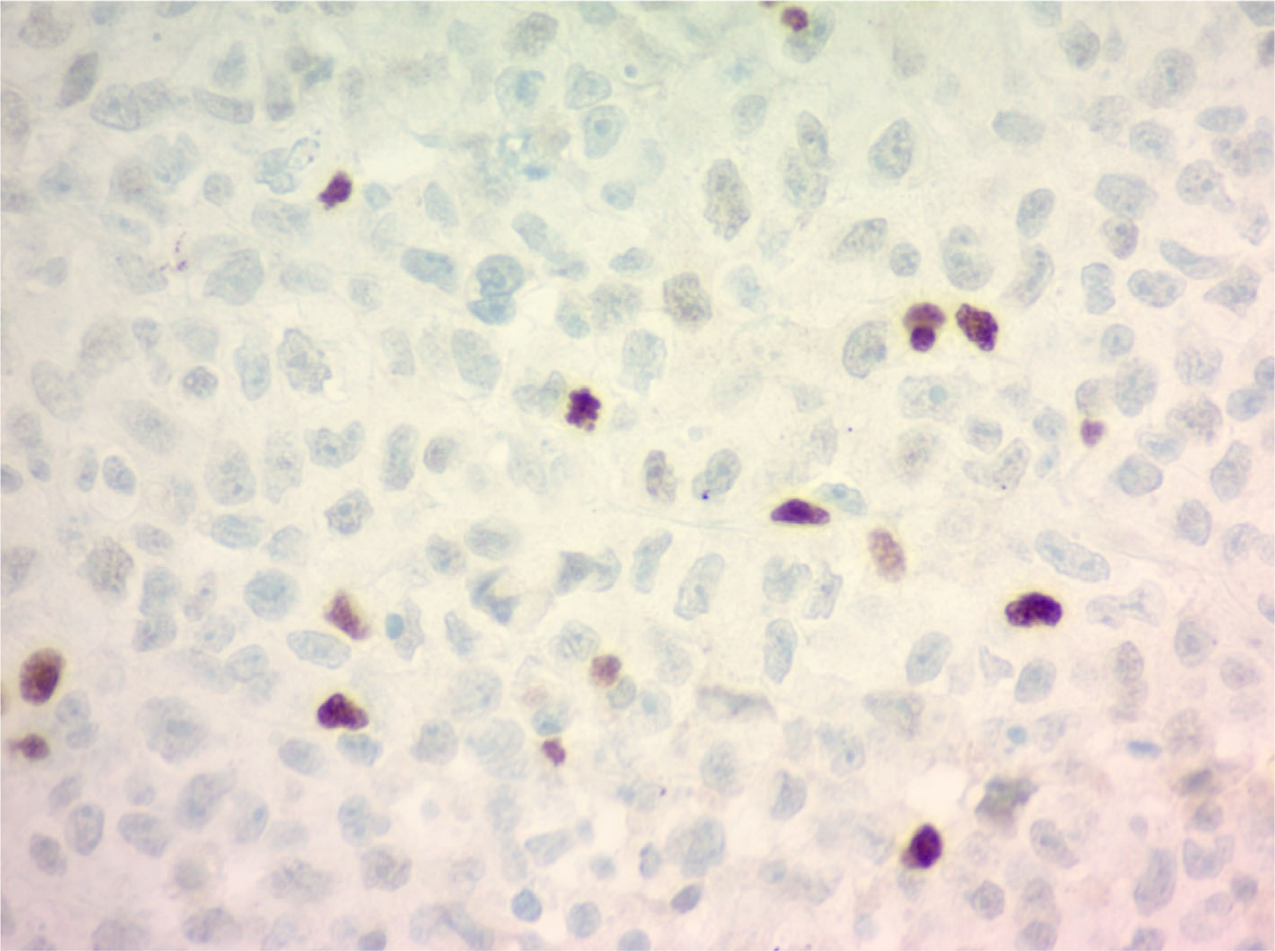
Immunostain for SMARCA4, with most tumor cells showing complete loss of SMARCA4 staining. (40×).

**FIGURE 4:**
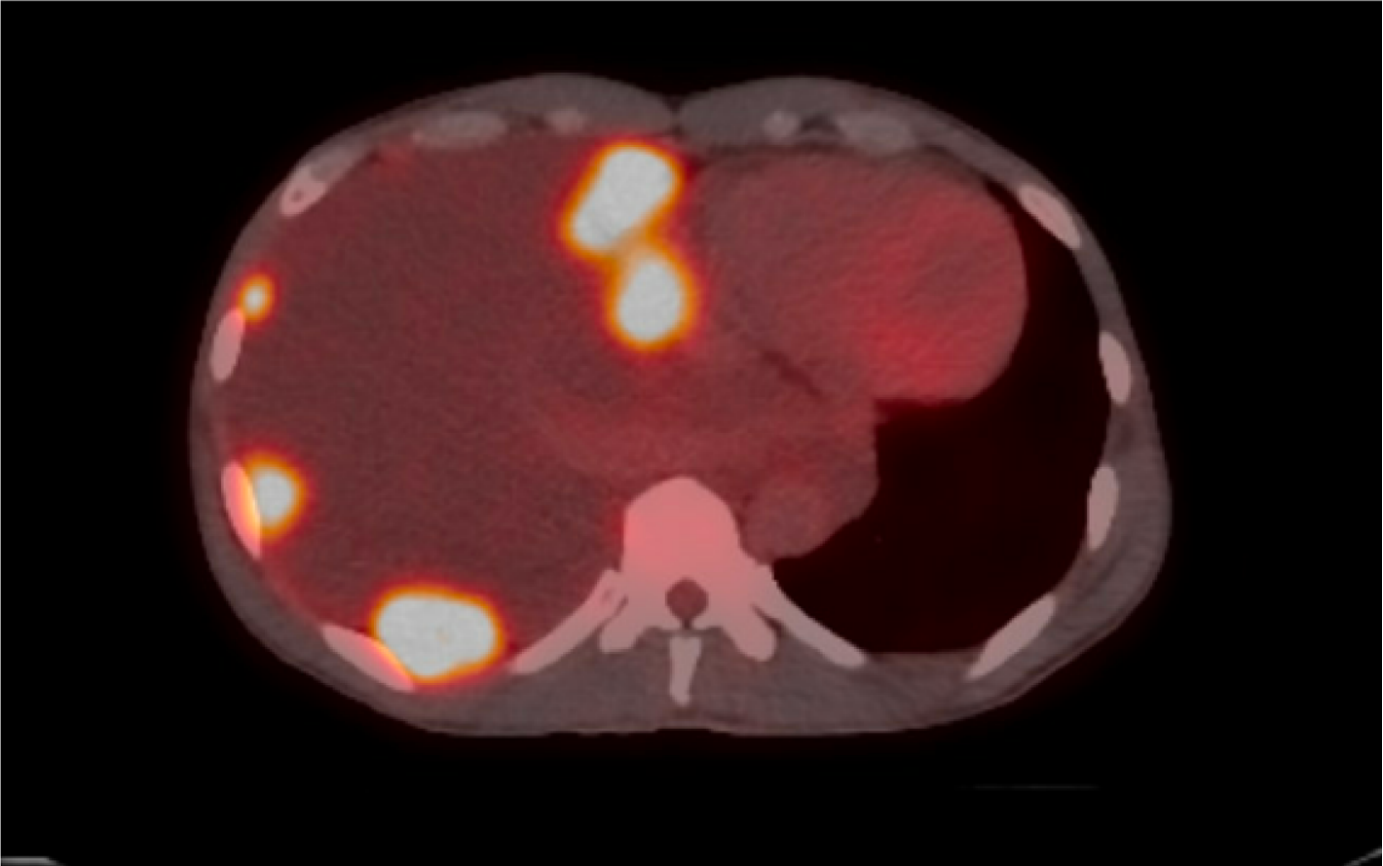
Positron emission tomography/computed tomography (PET/CT) showed innumerable FDG‐avid right pleural‐based lesions with a maximum standardized uptake value (SUV) of 14.3, as well as fluorodeoxyglucose (FDG)‐avid right hilar and bilateral mediastinal adenopathy.

A 40‐year‐old male with a 20 pack‐year tobacco history, work‐related dust and melted nickel exposure, and marijuana use was admitted with a history of several weeks of dyspnea. He had no known family history of lung disease or lung cancer. Computed tomography (CT) of the chest revealed numerous right‐sided pleural‐based masses and soft tissue plaques, a right pleural effusion, diffuse thoracic adenopathy and bilateral giant bullous paraseptal emphysema (Figure 1). Thoracentesis was not performed due to the high risk of bleeding from multiple pleural masses and a paraseptal bullous emphysema. A CT‐guided needle biopsy revealed undifferentiated non‐small cell lung cancer (NSCLC), with features of SMARCA4‐deficiency (Figures 2 and 3). Positron emission tomography (PET) scans showed innumerable flurodeoxyglucose (FDG)‐avid pleural, right hilar, and right mediastinal lesions (Figure 4). Next‐generation sequencing (NGS) showed mutations in BRCA1 at p.S1796 and a missense variant loss‐of‐function mutation in TP53 at p.V274A. The patient was prescribed six cycles of carboplatin and paclitaxel. A follow‐up CT scan showed stable findings with no evidence of disease beyond the right lung. Due to new shortness of breath, his treatment was changed to nivolumab since his tumor mutational burden (TMB) was 12.6. After one cycle, the patient presented with weakness. Magnetic resonance imaging (MRI) revealed a new 4.5 cm intraspinal mass affecting T6‐T7 and causing mid‐thoracic spinal cord compression. Neurosurgical resection confirmed SMARCA4‐deficient undifferentiated NSCLC that had spread to the spine. Continuing follow‐up treatment will be performed on an outpatient basis.

## DISCUSSION

SMARCA4‐UT are difficult to detect and have a poor prognosis. Diagnostic biopsy can be dangerous due to typically extensive extant tissue destruction that could lead to biopsy‐induced pneumothorax. This scarcity of tissue makes it difficult to perform extensive pathological testing.^8^ Interpretation of imaging is arduous due to wide radiological variation coupled with the extensive tissue destruction usually found upon diagnosis.^9^

The patient in this case study was treated with carboplatin and paclitaxel, since case reports suggest a progression‐free survival of approximately 6 months or longer with treatment.^10^ The decision to use second‐line nivolumab was based on a few case reports that have shown remarkable response, with one case reporting survival for more than 3 years.^11^, ^12^, ^13^ NGS can be helpful in identifying mutations that can be targeted. Even though our patient had a BRCA1 mutation that may benefit from treatment with a PARP inhibitor, there are no current trials suggesting this course.

Further investigation into the pathogenesis of SMARCA4‐UT may identify additional treatment options for this highly fatal entity. SMARCA4 is an ATP‐dependent chromatin remodeling protein, which combines with the Brahma‐related gene (BRG) to form a catalytic subunit known as the switch/sucrose (SWI/SNF) nonfermentable complex. Mutations of SMARCA4 result in inactivation and may cause a variety of cancers in different organ systems. An activating mutation of catenin beta‐1 (CTNNB1) and loss‐of‐function mutation of SMARCA4 that occur concurrently also appear to cause tumorogenesis.^14^ This relationship between CTNNNB 1 and SMARCA‐4 UT is currently under investigation as a potential future treatment target. A retrospective cohort study from 2020 showed that SMARCA4‐deficient thoracic tumor patients treated with a PD1/PD‐L1 inhibitor may achieve at least some partial response.^15^ Other reports show potential for future drugs like ataxia telangiectasia and Rad3‐related (ATR) inhibitors to target DNA damage repair of the cancer cells.^16^ Furthermore, inhibitors such as the Aurora kinase A (AURKA) inhibitor could be future targets since they destroy the mitochondrial oxidative phosphorylation of those respective cancer cells.^17^

SMARCA4‐UT thoracic tumors are rare but aggressive forms of cancers. SMARCA4‐deficiency leads to tumorigenesis within several organ systems. Due to the rarity of this condition, prompt diagnosis can often be challenging. Treatment is also unclear in SMARCA4‐UT related cancers; however, clinical trials with novel inhibitors are anticipated for the future. Our hope is with the presentation of this particular case with SMARCA4‐UT thoracic cancer, we can add to the library of existing literature for this disease process.

## AUTHOR CONTRIBUTIONS

Dr. Hanona organized theclinical information, provided an in depth analysis, and wrote themanuscript. Dr. Ezekwudo helped toorganize the clinical information and lead the patient's case. Dr. Fullmer provided pathological pictures aswell as captions. Dr. Allen alsoprovided pathological pictures as well as captions. Dr. Jaiyesimi supervised the writing of themanuscript.

## FUNDING INFORMATION

This study did not receive any funding from the public, private or not‐for‐profit sectors.

## CONFLICT OF INTEREST STATEMENT

The authors declare no conflicts of interest.

## Data Availability

No databases were involved in the preparation of this manuscript.
